# Supplementary material for: A Current State of Proteomics in Adult and Pediatric Inflammatory Bowel Diseases: A Systematic Search and Review
Source: Int J Mol Sci. 2023 May 27;24(11):9386. doi: 10.3390/ijms24119386 (PMC10253608; doi:10.3390/ijms24119386)
Supplement: Supplementary file 1 [file ijms-24-09386-s001.zip › ijms-2393490-supplementary.pdf]

Supplementary Table S1: A list of methods used for data analysis in the selected studies.

Reference	Data analysis
Meuwis MA et al., 2007 [40]	A decision tree boosting algorithm was used for data analysis.<br>Leave-one-out cross-validation was applied for an unbiased estimation of the sensitivity and specificity of a classification.<br>Biomarkers were evaluated individually by univariate and multivariate analysis.<br>The discriminative power of peaks was assessed by non-parametric Mann-Whitney test.<br>Correlation coefficient (r) and associated P value were determined with the non-parametric Spearman test and by Mann–Whitney U test.
Shkoda A et al., 2007 [42]	The difference in medians between two groups of observations was assessed by nonparametric Mann-Whitney U Test.<br>For multiple test correction, the Bonferroni test was used.
Meuwis MA et al., 2008 [41]	Biomarker Wizard software was used for spectral peak integration.<br>Peak distribution was analyzed by univariate method performing analysis of variance test.<br>A multivariate analysis based on supervised classification with multiple decision trees boosting method was also used.<br>A Mann-Whitney U test was used to compare each patient before and after infliximab therapy.<br>Correlations were calculated by Spearman test and Wilcoxon paired tests.<br>A Fisher's exact test was used in the analysis of contingency tables.
Brentnall TA et al., 2009 [43]	The mass spectrometry data were processed using Trans-Proteomic Pipeline and then searched against IPI human protein database using the SEQUESTR algorithm.<br>All the peptide identifications were validated using PeptideProphet.<br>The iTRAQ quantification was achieved using LIBRA program.<br>MetaCore was used to map the differentially expressed proteins into biological networks.<br>The biological process enrichment was analyzed based on Gene Ontology processes.<br>The Analyze Network algorithm was used to map protein pathways within two-step interactions.
Kanmura S et al., 2009 [44]	Statistical analyses were performed using StatView 4.5 software.<br>Statistical significance was determined using Mann–Whitney U and paired t-tests.<br>The discriminatory power for each marker was described via the area under the curve from receiver operating characteristic analysis.
M'Koma AE et al., 2011 [45]	Raw peptide spectra were analyzed by Biodesix software.<br>Pair-wise statistics was performed for comparisons of the sample subsets.
May D et al., 2011 [46]	Raw output files from all mass spectrometry runs were searched with X!Tandem software against the human International Protein Index database.<br>Peptide identifications were assigned probability by PeptideProphet software.<br>Statistical analysis of the immunohistochemical results was performed using GraphPad Prism.<br>For peptide abundance comparison between the groups, a t-test was used.<br>A false discovery rate analysis was performed for multiple testing correction.<br>Differences in the biomarker level between non-progressors and progressors were tested using the Mann-Whitney U test.

	Empirical receiver operating characteristic curves were used to determine the sensitivity and specificity of the biomarker.
Zhao X et al., 2011 [47]	The differences between two groups were assessed by two-sample t-test.<br>A Bonferroni approach was used for multiple testing correction.<br>A Pearson chi-square test was used to analyze the associations between protein expression and pathological features.<br>A Fisher exact test was used in the analysis of contingency tables.<br>The differences in cytokine expression were compared by unpaired Student t-test.<br>Bartlett test verified the equal variances across the groups.
Poulsen NA et al., 2012 [48]	A t-test was used to identify spots with significantly different protein levels in the inflamed versus non-flamed mucosa.<br>A false discovery rate analysis was performed for multiple testing correction.<br>Multivariate statistics was applied for 2D gel electrophoresis datasets analysis.<br>The normalized spot volumes were analyzed in SIMCA 9.0 software.<br>Peptide mass fingerprinting protein searches were conducted in the Mascot ions search engine using the Swiss-Prot database.
Seeley EH et al., 2013 [49]	Spectra were statistically compared in ClinProTools software.<br>A table of Wilcoxon rank sum p-values was generated and sorted to determine the proteins showing the greatest difference between the classes.<br>Receiver operator curves were generated for each peak.<br>A Support Vector Machine algorithm was generated using all of the data from actively inflamed areas within the mucosa.
Zhou Z et al., 2013 [50]	Mass spectrometry data were compared against tryptic peptide sequences from the SWISS-PROT database using Mascot algorithms.<br>Western blot analysis data were analyzed by Quantity One software.
Han NY et al., 2013 [51]	A MetaCore 6.4 software was used to map the differentially expressed proteins into biological networks.<br>A network analysis algorithm was used to map the protein pathways within two-step interactions.
Gazouli M et al., 2013 [52]	A Kolmogorov–Smirnov/Lillie for test was used to check the normal distribution of the values.<br>Normally distributed data were compared by two-pair t-test.<br>Means of spot intensities for proteins with not normally-distributed values were compared for statistical significance with the Mann–Whitney nonparametric test.<br>A false discovery rate analysis was performed for multiple testing correction.
Vaiopoulou A et al., 2015 [53]	A statistical software GraphPad Instat 3 was used for the analysis.<br>Means of spot intensities for proteins with not normally-distributed values were compared for statistical significance with the Mann–Whitney nonparametric test.<br>A false discovery rate analysis was performed for multiple testing correction.<br>Optical density means of the bands for each protein in Western blot analysis were compared with two sample t-test.
Bennike TB et al., 2015 [54]	Two-side t-test was performed to identify proteins with a statistically significant mean abundance change between the ulcerative colitis group and the control group.<br>Permutation-based false-positive control was applied to correct for multiple hypothesis testing.<br>Proteins displaying a statistically significant mean abundance change were further investigated using SPSS statistics v.22.

Townsend P et al., 2015 [55]	Protein identifications were performed using MSGF+ search engine against Uniprot database of human protein sequences and the decoy database.<br>A false discovery rate analysis was performed for multiple testing correction.<br>iTRAQ reporter ion intensities for identified peptides were extracted using MASIC software.<br>Peptide IDs and abundances in each sample were statistically analyzed in MatLab.<br>Peptides with a significant quantitative difference were calculated using repeated measures of analysis of variance with a Tukey post-hoc test.<br>Partial least squares-discriminant analysis was used as a supervised learning approach to evaluate the classification potential among the disease groups.<br>The biological functions and the roles of selected proteins in disease were derived from Uniprot.
Corfe BM et al., 2015 [56]	Phenyx, Uniprot and Progenesis QI software was used for the analysis of identified peptides.<br>Differences between groups were evaluated with the nonparametric Mann-Whitney U test.<br>A Bonferroni approach was used for multiple testing correction.
Starr AE et al., 2017 [57]	Mass spectrometry results were analyzed using MaxQuant v1.5.1 software.<br>Mathematical models for disease classification were developed in Receiving Operating Characteristics Curve Explorer and Tester using proteomic data from discovery cohort.<br>Candidate biomarkers were ranked by the respective area under the receiver operator curve value.<br>The strength of the relation between the selected variables was measured by Pearson correlation.
Stidham WR et al., 2017 [58]	All analyses were performed using SAS 9.4.<br>Demographic, clinical, and laboratory features were compared between inflammatory and fibrostenotic groups using the Student t-test and a Chi-squared or Fisher exact test.<br>Protein abundance, relative to internal standards, was compared between the inflammatory and fibrotic state using a two-sample t-test.<br>A false discovery rate analysis was performed for multiple testing correction.<br>The sensitivity, specificity, and area under the receiver operating characteristic curve were reported for proteins able to be detected by enzyme-linked immunosorbent assay using logistic regression.
Moriggi M et al., 2017 [59]	Statistical analysis was performed using the DeCyder 1.0 extended data analysis module.<br>For proteomic experiments, statistically significant differences were computed by independent one-way analysis of variance test, coupled to Tukey multiple group comparison test.<br>A false discovery rate analysis was performed for multiple testing correction.<br>A Student t-test was used to compare differences between two groups.
Ning L et al., 2019 [60]	Mass spectrometry results were analyzed using MaxQuant v1.5.1 software and searched against the human UniProtKB database.<br>ProteinAtlas was used to analyze the tissue specificity of proteins differentially expressed between patients with inflammatory bowel diseases and the controls.<br>Multivariate principal component analysis was used to summarize sample classification on the basis of expression profiles of all proteins.<br>A GraphPad Prism 6 software was used for the statistical analyses.<br>Unpaired Student t-test was used to compare differences between two groups.

	For more than 2 groups, analysis of variance was used.
Erdmann P et al., 2019 [61]	Statistical analysis was performed using GraphPad Prism Software.<br>Statistical significance of differences in mRNA and protein expression between inflamed/noninflamed biopsy specimens and control samples was determined by performing a Kruskal-Wallis nonparametric analysis of variance with Dunn multiple comparison test.<br>For paired biopsy analysis, sample differences were evaluated using a Wilcoxon matched-pair test.<br>The Jonckheere-Terpstra trend test was applied to confirm significant tendencies in changes of gene expression and protein abundance.<br>Potential interaction between microRNA/cytokine expression and mRNA expression of targeted genes was analyzed by performing a Spearman rank correlation test.
Schniers A et al., 2019 [62]	Mass spectrometry results were analyzed using MaxQuant software against a UniProt fasta file of all human proteins.<br>Further analyses were performed in Perseus and Cytoscape/ClueGO.<br>The ulcerative colitis and heathy patients' groups containing separate values for each sample were compared to each other with a two-sample test.<br>Proteins were annotated with gene ontology terms from the respective online databases.
Pierre N et al., 2020 [63]	Identifications and quantifications of proteins were obtained using the MaxQuant software v. 1.5.5.110 and searched against the Uniprot-human database.<br>The enrichment pathway analyses were done using the KEGG pathway databases.<br>Statistical analyses were done using Perseus software v. 1.6.0.7 and GraphPad Prism v. 7.0.<br>Differences between protein abundances were assessed using a two-tailed paired t-test.<br>To correct multiple testing error, a Benjamini-Hochberg method was performed.<br>In the histological experiment, normality of the distribution has been tested with the Shapiro-Wilk test. When appropriate, two-tailed paired t-test or two-tailed Wilcoxon matched-pairs signed-ranks test was applied.
Arafah K et al., 2020 [64]	Mass spectrometry results were analyzed using MaxQuant v1.5.2.8 software and Andromeda search engine.<br>Perseus version 1.5.0.15 was used for statistical assessment.<br>Two-sample t-tests were used to identify the significantly more abundant proteins in one group compared to the other.<br>A false discovery rate analysis was performed for multiple testing correction.
Merli AM et al., 2020 [65]	The analysis of the spectra for the protein identification was performed using MaxQuant v. 1.5.2.8.<br>A one-way analysis of variance test was used to compare the three groups and a paired Student t-test to compare groups two by two.<br>A GraphPad Prism 7.00 was used for statistical analyses of immunohistochemical results.<br>Kruskal-Wallis test was applied to compare biomarker staining intensity score in all groups.<br>Wilcoxon paired or Mann-Whitney unpaired tests were applied when appropriated to compare groups 2 by 2.

	A Chi-squared test was used to compare all groups and Fisher exact test for 2 by 2 comparisons for the biomarker staining pattern human analysis.<br>A one-way analysis of variance test was used to compare the percentage of Ki67 positive cells in all groups.<br>Turkey post-hoc test was applied to compare groups 2 by 2.<br>Pearson r correlation test was applied to analyze the correlation between biomarker staining intensity score and the percentage of Ki67 positive cells.<br>Chi-square or Fisher exact tests were applied when appropriate to test the association of biomarker staining pattern with different Ki67 staining categories.
Pierre N et al., 2020 [66]	For each protein, an optimal cut-off was identified as the one maximizing the Youden index.<br>An association of each protein with time to relapse was assessed with the univariate Cox model.<br>The survival curves were estimated using the Kaplan-Meier method and they were compared with the logrank test.<br>To correct multiple testing error, a Benjamini-Hochberg method was performed.<br>The proportional hazard assumption was checked using both the Schoenfeld residuals test and graphically by verifying whether the survival curves crossed.<br>The strength of association between two variables was evaluated with the Spearman rank correlation.<br>Survival analyses and volcano plots were generated using lifelines Python library and ggplot2 R package.
Liu L et al., 2022 [67]	The identified proteins were annotated using the UniProt-GOA database.<br>Based on the information in the KEGG database, the protein pathways have been<br>determined.<br>For protein-protein interactions, a search was performed against the STRING database version 11.0.<br>Statistical analysis of the data was performed using the statistical software package R-3.4.3, Empower-Stats, and GraphPad Prism 7.<br>Differences between groups were analyzed using Student's t-test, the Mann–Whitney U test, and the Wilcoxon signed-rank test.<br>In case of continuous variables, the Kruskal–Wallis rank sum test was performed; if the count variable had a theoretical number <10, Fisher exact probability test was applied.<br>Pearson correlation coefficient, principal component analysis, and relative standard deviation were used to evaluate the repeatability of protein quantification.
Gruver AM et al., 2022 [68]	Statistical analysis was performed using the statistical software package R.<br>To correct multiple testing error, a Benjamini-Hochberg method was performed.
Vessby J et al., 2022 [69]	Mass spectrometry results were analyzed using MaxQuant software.<br>All statistical analyses were performed using R.<br>Patient characteristics were compared using the Wilcoxon–Mann–Whitney or Kruskal–Wallis test for continuous variables and the Pearson χ^2 test for discrete variables.<br>A Wilcoxon–Mann–Whitney test was also performed for each protein to confirm that findings in the unadjusted linear models were not heavily influenced by skewed distributions.

	The variable importance was computed as mean decrease in the Gini index.<br>The cohort size for the validation step was calculated based on a t-test.<br>For multiple test correction, the Bonferroni test was used.<br>Meta-analysis was performed using the R "metafor" package assuming a random effects model.<br>Differences in immunohistochemical intensity were compared using the Wilcoxon–Mann–Whitney test.
Louis Sam Titus ASC et al., 2022 [70]	GraphPad Prism 7, Microsoft Excel, easyROC software, and RStudio were used to plot and analyze biomarker data.<br>Comparisons of biomarker groups were analyzed using Mann–Whitney U test.<br>Correlation analysis was performed using the Spearman and Pearson methods.<br>For sensitivity, specificity, positive predictive value, negative predicative value, and area under the receiver operating characteristic curve analysis, the easyROC software was utilized.<br>To analyze significant differences between the groups tested, a nonparametric Mann–Whitney-U-test was used.

Supplementary Table S2: A full list of proteomic studies in human IBD

Reference	Type of the tissue	Cohort (n)	Proteomic technique	Main findings
Hsieh SY et al., 2006 [71]	Intestinal samples	UC (4), infectious colitis (3), HC (5)	2-DE and MALDI-TOF-MS	Identification of 13 down-regulated and 6 up-regulated proteins in patients with UC. Eight of the down-regulated proteins were mitochondrial (HSPA9B, HSPD1, ATP5B, PHB, MDH2, VDAC1, PRDX1 and PRDX2), suggesting the enterocyte mitochondrial dysfunction involvement in pathogenesis of UC.
Meuwis MA et al., 2007 [40]	Serum	CD (30), UC (30), inflammatory control (30), HC (30)	SELDI-TOF-MS	Identification of four proteins (PF4, MRP8, FIBA and Hpalpha2) that could serve as potential biomarkers of IBD.
Berndt U et al., 2007 [72]	Intestinal samples	CD (10), UC (10) HC (10)	MELC IF with specific antibody	Analysis of T lymphocyte subpopulations in the intestinal mucosa of IBD patients with distinctive phenotypic characteristics.
Shkoda A et al., 2007 [42]	Isolated intestinal epithelia	CD (6), UC (6), CRC (6)	2-DE and MALDI-TOF-MS	Identification of protein panels involved in signal transduction, stress response and energy metabolism that are up-

				regulated in IBD patients compared to controls.
Nanni P et al., 2007 [73]	Serum	CD (15), UC (26), HC (22)	Solid-phase bulk protein extraction and MALDI-TOF-MS	Detected signals were able to distinguish between the cohorts with 96,9 % prediction ability.
Fogt F et al., 2008 [74]	Intestinal samples	UC (5)	2-DE and LC-MS/MS	Identification of several proteins (protocadherins, α-1-antitrypsin, caldesmon and tetratricopeptide repeat domains) involved in inflammation and tissue repair in the inflamed colonic mucosa. Detection of mutated desmin as a potential biomarker of UC.
Meuwis MA et al., 2008 [41]	Serum	CD (20)	SELDI-TOF-MS	Higher levels of PF4 were found in non-responders to infliximab therapy.
Nanni P et al., 2009 [75]	Serum	CD (15), HC (48)	Label-free ESI/Q-TOF-MS	Identification of serum proteins such as FIBA, FPA or C3f showing exoprotease activity characteristic for patients with CD.
Nanni P et al., 2009 [76]	Isolated intestinal epithelia	CD (2), HC (2)	Label-free LC-TOF-MS and targeted MS/MS	Identification of several membrane, cytosolic and nuclear proteins (heat shock 70 kDa protein 5, tryptase alpha-1 precursor, ubiquitin, annexin A1 and others) up-regulated in CD patients compared to controls.
Brentnall TA et al., 2009 [43]	Intestinal samples	UC (15), HC (5)	iTRAQ and HPLC-TOF-MS/MS	Several proteins involved in UC neoplastic progression including those related to mitochondria, oxidative activity and calcium-binding were identified. Two of them (CPS1 and S100P) were further confirmed by the immunohistochemistry.
Kanmura S et al., 2009 [44]	Serum	CD (22), UC (48), CRC (5), infectious colitis (6), HC (13)	SELDI-TOF-MS	Human neutrophil peptides 1, 2 and 3 are significantly higher in

				patients with active UC compared to patients with UC in remission or other diseases and decrease after successful corticosteroid therapy. The proteins may serve as biomarkers of active UC and predict treatment outcomes.
Hatsugai M et al., 2010 [77]	Peripheral blood mononuclear cells	UC (17), CD (13), HC (17)	2-DE and MALDI-TOF-MS/MS	Identification of panel of proteins involved in inflammation, oxidation/reduction, cytoskeleton, endocytotic trafficking and transcription that could discriminate between CD and UC patients.
M'Koma AE et al., 2011 [45]	Intestinal samples	Colonic CD (24), UC (27)	Histology-directed MALDI-MS	Distinctive spectral peaks in submucosal layer were able to discriminate between colonic CD and UC.
May D et al., 2011 [46]	Intestinal samples	UC (15)	Label-free LTQ/Orbitrap hybrid MS coupled with nano-flow HPLC	Identification of several protein clusters differentially expressed in dysplastic and non-dysplastic mucosal regions in patients with UC-associated dysplasia or cancer. Two proteins (TRAP1 and CPS1) were detected in both dysplastic and non-dysplastic tissue by immunohistochemistry.
Zhao X et al., 2011 [47]	Intestinal samples	UC (12), HC (12)	2-DE and MALDI-TOF-MS	Increased expression of P-p38 and decrease of MAWBP and galektin-3 were found in UC patients compared to controls and correlated with disease progression. P38 MAPK pathway is suggested to be involved in patients with active UC.

Li N et al., 2012 [78]	Intestinal samples	UC (30), HC (30)	2-DE and MALDI-TOF-MS	Identification of 9 proteins related to hormonal modulation, immune response, oxidative stress and signal conduction differentially expressed in ulcerative and normal tissue. Six proteins were up-regulated (apolipoprotein C-III, haptoglobin, receptor tyrosine kinase, aldehyde reductase, pericentriolar material 1 and heat shock factor protein 2) and 3 were down-regulated (keratin, filamin A-interacting protein 1 and tropomyosin 3).
Poulsen NA et al., 2012 [48]	Intestinal samples	UC (20), HC (4)	2-DE and MALDI-TOF-MS	Forty-three proteins differentially expressed in inflamed colonic tissue in UC patients were identified. The proteins were mainly involved in energy metabolism (triosephosphate isomerase, glycerol-3-phosphate-dehydrogenase, alpha enolase and L-lactate dehydrogenase B-chain), and oxidative stress (superoxide dismutase, thioredoxins and selenium binding protein).
Erickson AR et al., 2012 [79]	Stool	6 twin pairs with either colonic CD, ileal CD or HC	2-DE and LC-MS/MS	Genomic, proteomic and microbiome analysis of the cohorts. Identification of protein a gene profiles distinguishing ileal CD from HC.
Presley LL et al., 2012 [80]	Intestinal mucosal-luminal interface	cohort 1: UC (6), HC (3)<br>cohort 2: CD (14), UC (15), HC (13)	SELDI-TOF-MS and MALDI-TOF-MS analysis of metaproteome	35 % of the gut bacteria differentiated patients by disease type.
Kwon SC et al., 2012 [81]	Intestinal samples	UC (6), tuberculous colitis (6), HC (6)	2-DE and MALDI-TOF-MS	Three proteins (mutant β-actin, α-enolase and Charcot-Leyden crystal

				protein) showed altered expression in TC and UC patients compared to normal tissue. Expression of α-enolase was higher in tuberculous colitis patients compared to normal tissue, but decreased in comparison with UC, representing a potential biomarker for differential diagnosis of tuberculous colitis and UC.
Seeley EH et al., 2013 [49]	Intestinal samples	Colonic CD (26), UC (26)	Histology-directed MALDI-MS	Based on 25 protein spectral peaks, a machine learning algorithm capable of differentiating between colonic CD and UC with 76,9 % accuracy was constructed.
Zhou Z et al., 2013 [50]	Intestinal samples	CD (8)	2-DE and MALDI-MS	Identification of 6 differentially expressed proteins in mucosal lesions compared to normal intestinal mucosa including prohibitin, calreticulin, apolipoprotein A-I, intelectin-1, protein disulfide isomerase and glutathione S transferase Pi.
Han NY et al., 2013 [51]	Intestinal samples	CD (3), UC (4), inflammatory polyps in UC (2), HC (3)	Label-free LC/MS	Identification of 3 proteins (PRG2, LCP1 and PSME1) serving as potential biomarkers of active CD.
Gazouli M et al., 2013 [52]	Serum	CD (18)	2-DE and MALDI-TOF-MS	Identification of a panel of proteins (APOA1, APOE, CO4B, PLMN, TRFE, APOH and CLUS) that are up-regulated in primary non-responders to infliximab therapy.
Kohashi M et al., 2014 [82]	Serum	CD (39), UC (120), HC (120)	GC/MS	Development of diagnostic models discriminating UC, CD and HC. The model differentiating UC from HC showed 93,33 % sensitivity and 95,00 %

				specificity in the training set and 95,00 % sensitivity and 98,33 % specificity in the validation set. The model discriminating UC from CD showed 85,00 % sensitivity, and 97,44 % specificity in the training set and 83,33 % sensitivity in the validation set. The model assessing UC showed 84,62 % sensitivity and 88,23 % specificity in the training set, 84,62 % sensitivity and 91,18 % specificity in the validation set and correlated with the clinical activity index.
Vaiopoulou A et al., 2015 [53]	Serum	CD (12), pCD (12)	2-DE and MALDI-TOF-MS	Ceruloplasmin and apolipoprotein B-100 are increased in children with CD, while clusterin is overexpressed in adult CD patients.
Piras C et al., 2014 [83]	Serum	CD (44), HC (13)	2-DE and MALDI-TOF-MS	Over-expression of several inflammatory proteins and complement 3 chain C and under-expression of clusterin, retinol binding protein, transthyretin and α-1-microglobulin in early-stage CD.
Miao J et al., 2014 [84]	Serum and intestinal samples	UC (40), CD (29), intestinal tuberculosis (31), intestinal lymphoma (32), Behçet's disease (28), infective enteritis (32), HC (50)	2-DE and MALDI-TOF-MS	Heat shock factor 2 was overexpressed in the intestinal mucosa in patients with UC compared to other cohorts. The expression correlated with the severity of the disease. The serum concentration of the protein also corelated with levels of IL-1β and TNFα.
Bennike TB et al., 2015 [54]	Intestinal samples	UC (10), HC (10)	Label-free LC/MS	Identification of 46 proteins differentially expressed in UC compared to controls. The proteins were often associated with

				neutrophils and neutrophil extracellular traps formation suggesting involvement of the innate immunity.
Townsend P et al., 2015 [55]	Serum	Stricturing CD (9), non-stricturing CD (9), UC (9)	LC-MS	Identification of the peptide/protein subset discriminating between the three cohorts with 70 % accuracy for peptides and up to 80 % for proteins. Several proteins distinguishing the stricturing CD were involved in complement activation, fibrinolysis and lymphocyte adhesion.
Corfe BM et al., 2015 [56]	Intestinal samples	UC (45), PSC-UC (7), HC (10)	iTRAQ and HPLC-ESI/Q-TOF-MS/MS	Down-regulation of keratins 8, 18 a 19 and vimentin in patients with acute distal UC compared to controls and samples from non-inflamed proximal mucosa. Up-regulation of those proteins in patients with quiescent longstanding pancolitis in contrast to controls and patients with recent-onset remission. Decreased levels of several intermediate filament proteins in patients with PSC-UC and UC with dysplasia.
Wasinger VC et al., 2016 [85]	Serum	Discovery phase: CD (20), UC (20), HC (10)<br>Validation phase: CD (56), UC (27), HC (14), rheumatoid arthritis (12)	Label-free LC-MS/MS	Secreted phosphoprotein 24 (SPP24) differentiated IBD patients from HC. UC patients in remission from HC were distinguished by SPP24 and α-1-microglobulin and CD patients in remission from HC by SPP24. Guanylin and secretogranin-1 discriminated between CD and UC.
Zhang F et al., 2016 [86]	Serum	CD (30), intestinal tuberculosis (21), HC (30)	Weak cation magnetic beads and MALDI-TOF-MS	Several diagnostic models of distinctive protein peaks were constructed that differentiated CD

				and HC with 96,7 % sensitivity and specificity, intestinal tuberculosis and HC with 93,3 % sensitivity and 95,2 % specificity and CD from intestinal tuberculosis with 76,2 % sensitivity and 80,0 % specificity respectively.
Heier CR et al., 2016 [87]	Serum	pCD (17), pUC (2)	Aptamer-based SOMAscan proteomics	Identification of 18 proteins and 3 miRNAs responsive to prednisone and infliximab treatment. Six markers appeared to be steroid specific. Eight increased markers were associated with inflammation and their promoters were regulated by NF-κB.
Li X et al., 2016 [88]	Intestinal mucosal-luminal interface	CD (21), UC (13), HC (17)	MALDI or HPLC-LC-MS/MS	Identification of protein modules differentially expressed throughout the gastrointestinal tract. Seven of the modules showed the association with respective patient cohorts.
Starr AE et al., 2017 [57]	Intestinal samples	Discovery cohort: pCD (15), pUC (15), pHC (20)<br>Validation cohort: pCD (15), pUC (15), pHC (19)	SILAC and HPLC-ESI-MS/MS	Two protein panels were identified that discriminate IBD from HC (FABP5, NAMPT, UGHD, LRPPRC and PPA1) and CD from UC (HADHB, SEC61A1, SND1, LAP3, LTA4H, MT2A, SLC25A1, HNRNP H3, TF, ECH1, TFRC and B2M), respectively. Application of the two panels to validation cohort differentiated 95,9 % IBD patients from HC and 80 % CD from UC. Hundred and sixteen proteins correlated with the severity of the disease. Visfatin and metallothionein-2 were subsequently confirmed

				by ELISA on independent cohort.
Di Narzo AF et al., 2017 [89]	Serum	CD (84), UC (88), HC (15)	SOMAmer-based capture array	Analysis of the genotype-protein associations and age dependent proteomic changes in IBD patients.
Stidham WR et al., 2017 [58]	Serum	CD (40)	LC-MS	Identification of 5 glycoproteins showing ≥ 20% abundance change in ≥ 80 % of the patients with inflammatory and fibrostenotic phenotype of CD. Among the glycoproteins, COMP and HGFA were elevated in the fibrostenotic group.
Moriggi M et al., 2017 [59]	Intestinal samples and isolated intestinal epithelia	CD (30), UC (30), controls (16)	2-DE and MALDI-TOF-MS	Identification of proteins increased or decreased in inflamed and non-inflamed IBD patients reflecting different patterns of extracellular matrix and cytoskeleton rearrangement and changes in cellular metabolism and autophagy.
Deeke SA et al., 2018 [90]	Intestinal mucosal-luminal interface	pCD (22), pUC (20), HC (18)	SILAC and LC-MS	Identification of 4 proteins discriminating active pIBD from non-IBD patients with sensitivity of 95,4 % and > 99,9 % for the ascending and descending colon respectively, and specificity of > 99,9 % for both ascending and descending colon. Another panel of 4 proteins distinguished pancolitic from non-pancolitic pUC patients with sensitivity and specificity > 99,9 %. The elevation of 2 proteins (catalase and LTA4H) in pIBD patients was confirmed in stool.
Denadai-Sousa A et al., 2018 [91]	Intestinal samples	CD (11), UC (11), controls (16)	Functional MS analysis using activity-based probes	Identification of 7 active proteases (cathepsin G, plasma kallikrein, plasmin, tryptase,

				chymotrypsin-like elastase 3 A, thrombin and the aminopeptidase B) from which cathepsin G and thrombin were overreactive in IBD patients compared to controls.
Di Narzo AF et al., 2019 [92]	Serum	CD (126), UC (46), HC (72)	SOMAmer-based capture array	Identification of a protein spectrum differentially expressed in IBD patients and controls. Up-regulated proteins showed association with immunity functionality, whereas down-regulated proteins were involved in nutrition and metabolism. The proteomic profiles were very similar in CD and UC patients. Plasma protein levels poorly correlated with blood and intestinal transcriptomes.
Jin L et al., 2019 [93]	Intestinal tissue	CD (9), UC (15), HC (10)	LC-MS/MS	Integrative proteomic and transcriptomic analysis in patients with IBD. In both CD and UC, dysregulation of genes and proteins involved in immune and inflammatory responses were found. CD patients demonstrated increased T-helper cell differentiation, elevation of JAK/STAT signaling and increased toll-like receptor expression. In UC, increased MAPK signaling was found.
Ning L et al., 2019 [60]	Intestinal samples	Discovery cohort: CD (9), UC (9), HC (6)<br>Validation cohort: CD (3), UC (3), HC (3)	LC-MS/MS	Identification of protein spectra differentially expressed in CD, UC and controls. Several novel proteins including CD38 were introduced. CD38 expression was higher IBD patients compared to controls, and in CD patients in comparison to UC. It was also more

				abundant in inflamed regions of the bowel.
Erdmann P et al., 2019 [61]	Intestinal samples	UC (10), HC (10)	LC-MS/MS	Identification of several genes for metabolizing enzymes (i.e., CYP2C9 or UGT1A1) and transporters (ABCB1, ABCG2 or MCT1) decreased during the inflammation. On the other hand, MRP4, OATP2B1 or PRCTL2 were significantly elevated in inflamed tissue. On the protein level, these results could be confirmed only for MCT1 though.
Schniers A et al., 2019 [62]	Intestinal samples	UC (17), HC (15)	LC-MS/MS	Down-regulation of several proteins including metallothioneins, PPAR-inducible proteins, fibrillar collagens and proteins involved in bile acid transport and metabolic functions of nutrients, energy, steroids, xenobiotics and carbonate in patients with UC. Up-regulated were proteins involved in immune response and protein processing in endoplasmic reticulum.
van der Post S et al., 2019 [94]	Mucus from colonic samples	UC (64), HC (47)	LC-MS/MS	Reduction of MUC2 protein in colon mucus barrier in patients with active UC. Active UC was associated with decreased numbers of sentinel goblet cells and attenuation of the goblet cell secretory response to microbial challenge. Mucus abnormalities may contribute to UC pathogenesis.
Pierre N et al., 2020 [63]	Intestinal samples	CD (16)	Label-free UPLC-ESI-MS/MS	Different proteins were expressed in ulcer edges from patients with ileal and colonic CD. The proteins were mainly

				associated with epithelial-mesenchymal transition, neutrophil degranulation and ribosomes.
Basso D et al., 2020 [95]	Stool	Discovery cohort: CD (72), UC (56), HC (34)<br>Validation cohort: CD (27), UC (15), HC (28)	LTQ-Orbitrap XL-MS and MALDI-TOF-MS	Identification of protein spectra associated with IBD diagnosis. Over-expressed proteins included immunoglobulins and neutrophilic proteins, under-expressed included nucleic acid assembly proteins or those related to cancer risk such as OLFM4 and ENPP7.
Arafah K et al., 2020 [64]	Intestinal samples	Discovery cohort: CD (9), UC (9)<br>Validation cohort: CD (62), UC (51)	LC-MS/MS	Identification of several proteins up-regulated in CD patients including proteins related to neutrophilic activity and damage-associated molecular patterns. Aldo-keto reductase family 1 member C3 protein was found in 8/9 CD patients and no UC patients.
Merli AM et al., 2020 [65]	Intestinal samples	Discovery cohort: UC (5)<br>Validation cohort: UC (74), sporadic lesions (174), HC (18)	Label-free LC-MS/MS	Proteomic analysis of dysplastic, inflammatory and normal mucosal regions in UC patients. Eleven proteins were found to be more abundant in dysplastic foci, including solute carrier family 12 member 2, which was subsequently confirmed by the immunohistochemistry.
Pierre N et al., 2020 [66]	Serum	CD (102)	UPLC-MS	Identification of several circulating biomarkers associated with risk of short-term and mid/long-term relapse after infliximab withdrawal.
Yuan X et al., 2021 [96]	Stool and serum	UC (129), non-UC patients with depression/anxiety (49), HC (62)	LC-MS	Analysis of intestinal microbiome diversity, metabolome and proteome interactions in UC patients with

				depression/anxiety in comparison to UC patients without depression/anxiety and controls.
Sun XL et al., 2021 [97]	Serum and intestinal samples	Discovery cohort: CD (20), HC (20) Validation cohort: CD (20), HC (20)	TMT and HPLC-MS	Fibrinogen-like protein 1 is up-regulated in CD patients and may trigger the intestinal inflammation by activation of NF-κB signaling pathway.
Park J et al., 2021 [98]	Intestinal samples	CD (47), Behçet's disease (47)	TMT and LC-MS/MS	Identification of 39 proteins differentially expressed in CD and intestinal Behçet's disease including beta-2 glycoprotein 1 and maltase-glucoamylase (MGAM). MGAM was increased also in serum of patients with BD.
Kalla R et al., 2021 [99]	Serum	CD (146), UC (153), IBDU (29), non-IBD (224)	Proximity extension assay	Identification of 66 proteins distinguishing IBD patients from symptomatic non-IBD controls, including matrix metallopeptidase-12 and oncostatin-M. Fifteen proteins, all members of tumor necrosis factor-independent pathways including interleukin-1 and oncostatin-M, predicted escalation of the therapy.
Liu L et al., 2022 [67]	Intestinal samples	UC (12)	Label-free LC-MS	Identification of 5 proteins (ACTBL2, MBL2, BIP, EIF3D and CR1) as potential predictive biomarkers of non-response to infliximab therapy.
Bourgonje AR et al., 2022 [100]	Serum	CD (188), UC (162)	Proximity extension assay	No protein signatures were found to reflect fatigue in patients with IBD.
Gruver AM et al., 2022 [68]	Intestinal samples	UC (19)	MS	Neutrophil-related proteins correlated with histological scoring indices of disease severity. A negative

				correlation was found between disease severity and cell junction proteins and β-catenin.
Vessby J et al., 2022 [69]	Intestinal samples	Discovery cohort: PSC-UC (9), UC (7), HC (7)<br>Validation cohort: (PSC-UC (16), UC (21)	LC-MS/MS	1-acetylglycerol-3-phosphate O-acyltransferase 1 was proved to be higher in PSC-UC patients compared to UC. The finding was confirmed by the immunohistochemistry.
Louis Sam Titus ASC et al., 2023 [70]	Serum	Discovery cohort: pUC (10), pCD (10), pHC (7)<br>Validation cohort: pCD (30), pUC (30), pHC (16)	Aptamer-based targeted proteomic assay	Significant elevation of serum resistin, elastase and lactoferrin in both pCD and pUC patients. The proteins (especially resistin) may serve as serum biomarkers of pIBD.
Alfredsson J et al., 2023 [101]	Isolated intestinal epithelial and immune cells	CD (3), CRC controls (4)	TMT and LC-MS	Mucosal immune cells of CD patients show increased proteins associated with neutrophil degranulation and mitochondrial metabolism, whereas epithelial cells show up-regulation of proteins involved in glycosylation and secretory pathways and down-regulation of those involved in mitochondrial metabolism.

2-DE = two-dimensional gel electrophoresis; CD = Crohn's disease; CRC = colorectal cancer; ESI/Q = electrospray ionization quadrupole; GC = gas chromatography; HC = healthy control; HPLC = high performance liquid chromatography; IBD = inflammatory bowel disease; IBDU = inflammatory bowel disease unclassified; IF = immunofluorescence; iTRAQ = isobaric tags for relative and absolute quantitation; LC = liquid chromatography; MALDI = matrix assisted laser desorption/ionization; MS = mass spectrometry; pCD = pediatric Crohn's disease; pHC = pediatric healthy control; PSC = primary sclerosing cholangitis; pUC = pediatric ulcerative colitis; SELDI = surface-enhanced laser desorption/ionization; SILAC = stable isotope labeling by amino acids in cell culture; TMT = tandem mass tag; TOF = time of flight; UC = ulcerative colitis; UPLC = ultra-high performance liquid chromatography.
